# A Comparison of the Geometrical Accuracy of Thin-Walled Elements Made of Different Aluminum Alloys

**DOI:** 10.3390/ma14237242

**Published:** 2021-11-27

**Authors:** Magdalena Zawada-Michałowska, Paweł Pieśko, Jerzy Józwik, Stanisław Legutko, Leon Kukiełka

**Affiliations:** 1 Department of Production Engineering, Faculty of Mechanical Engineering, Lublin University of Technology, 20-618 Lublin, Poland; p.piesko@pollub.pl (P.P.); j.jozwik@pollub.pl (J.J.); 2 Institute of Mechanical Technology, Faculty of Mechanical Engineering, Poznan University of Technology, 60-965 Poznan, Poland; stanislaw.legutko@put.poznan.pl; 3 Department of Mechanical Engineering, Faculty of Mechanical Engineering, Koszalin University of Technology, 75-453 Koszalin, Poland; leon.kukielka@tu.koszalin.pl

**Keywords:** geometrical accuracy, thin-walled elements, milling, HSC, aluminum alloys

## Abstract

In modern constructions, especially aircraft, the aim is to minimize the weight of the components used. This necessitates the use of innovative construction materials, or the production of these parts with ever-decreasing wall thicknesses. To simplify assembly and improve strength properties, so-called structural elements are being used in the form of monolithic elements, which are replacing the assemblies of parts joined by, for example, riveting. These structures often have a complex, thin-walled geometry with deep pockets. This paper attempts to assess the accuracy of manufacturing thin-walled elements, in the shape of walls with different geometries, made of various aluminum alloys. Machining tests were conducted at different cutting speeds, which allowed comparisons of the geometric accuracy of parts manufactured under conventional and high-speed cutting conditions. Based on the result obtained, it was found that the elements made of EN AW-7075 T651 alloy underwent the greatest deformations during machining in comparison to other two materials (EN AW-6082 T651 and EN AC-43000). An increase in the geometrical accuracy of the manufactured elements was also observed with the increase in the cutting speed for the HSC range. Hence, to minimize the postmachining deformation of thin-walled elements, the use of high-speed cutting is justified.

## 1. Introduction

**Figure 1:**
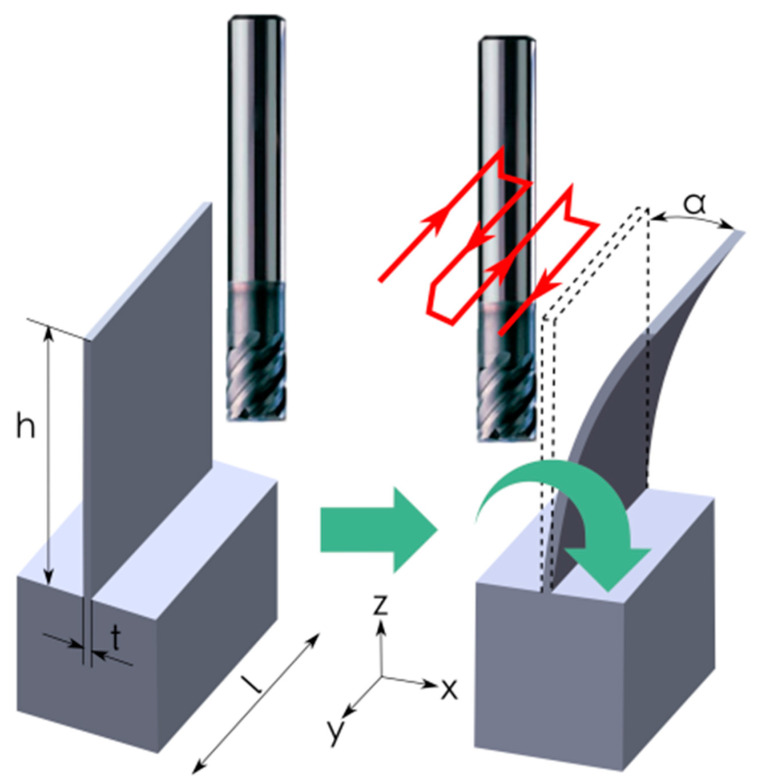
Deformation of thin wall during machining: *t*—wall thickness, *h*—wall height, *l*—wall length, *α*—angle of wall deflection.

Thin-walled pocket elements made of aluminum alloys are mainly used in the aircraft, automotive and shipbuilding industries, due to their low weight and high rigidity. Such structures are applied in aviation, among others, in the production of the fuselage, landing gear, transmission housings, and load-bearing components of aircraft. Other examples include frames, ribs, and girders. In the case of large-size structures, which mainly consist of many pockets, they are characterized by the ratio of height and length of the wall to its thickness of 10:1, 50:1, 50:1 and sometimes even 250:1 [1,2,3]. The major issues during machining this type of workpiece are its elastic and plastic deformations. When thin walls are milled, the upper part of the wall deflects under the pressure of the cutting edge as a result of elastic deformation. After the load is removed, the wall returns to its original position and assumes a characteristic trapezoidal outline in cross-section. Its thickness is smaller at the base and increases towards the upper edge of the wall. The value of the elastic deformation during machining depends on the mechanical properties of the materials being machined, i.e., in the considered case, the various aluminum alloys [4,5,6,7]. Figure 1 shows the deformation of a thin wall under the influence of the cutting force during machining.

Thin-walled elements are particularly susceptible to post-machining deformations, occurring mainly after the finished machining process. It is a very complex issue, and the formation of the above-mentioned deformations depends on many factors, including primarily the residual stresses generated at each stage of the technological process [8,9,10].

For the production of thin-walled pocket elements made of wrought aluminum alloys, rolled plates are usually used. They are characterized by anisotropy of structural and mechanical properties. It is the result of the accumulation of residual stresses being the effect of the technological history of the semifinished product. The balance of residual stresses is disturbed during machining and new stresses are introduced, and then they are translated into undesirable deformations and problems with obtaining appropriate dimensional and shape accuracy. The deformation of the machined thin-walled parts can also cause instability in the cutting process and contribute to the deterioration of the machined surfaces [8,11,12,13,14,15,16,17].

An important issue is also the fact that currently the materials are subject to very restrictive requirements, especially in aviation or automotive industries. Knowledge of the mechanical properties is therefore essential. Material characteristics depending on the studied variables are presented in numerous publications. In the case of thin-walled elements, it is important to understand the aforementioned properties, as well as the very mechanism of deformation formation [18,19,20].

The methods for reduction of residual stresses (e.g., heat treatment, seasoning, and vibration method) are widely used in industry. However, all the ways presented have weaknesses, such as decreases in production efficiency and energy losses, as well as generation of significant costs; therefore, efforts were made to eliminate these operations [14,21,22].

Difficulties during the machining of thin-walled components have necessitated the use of special cutting tools and machining strategies. Depending on the ratio of wall height to its thickness, one of the thin-wall machining methods can be used. They are based on different configurations of the alternating machining of both sides of the wall at successive levels, but these strategies do not have universal character [17,23,24,25]. However, the application of these strategies has some disadvantages:

- Repeated tool transitions introduce additional residual stresses, which can play a significant role in causing deformations in the machined walls;
- Making successive transitions along previous machining traces can cause a regenerative effect, i.e., a loss of stability in the machined wall or tool;
- During the machining of successive layers, the tool edge “rubs” against the previously machined surface, which accelerates tool wear and downgrades the quality of the machined surface.

There are also other methods of increasing the dimensional and shape accuracy of manufactured thin-walled elements by application of milling, e.g., increasing the cutting speed *v_c_* and rationalization of technological parameters, especially feed per tooth *f_z_* and milling width *a_e_*, reducing component of the cutting force perpendicular to the machined wall. It is also important that during milling of thin-walled elements, the contact time of the cutting edge with the workpiece should be limited. This is possible due to the use of increased cutting speeds *v_c_* and a small ratio of depth of cut *a_p_* to milling width *a_e_* (*a_p_*/*a_e_*) [17,26,27].

The paper [28] describes the possibility of minimizing deformations by applying the following solutions:

- Optimization of technological parameters, tool geometry and tool path;
- Simultaneous milling of both sides of the wall;
- Application of special ways for fixing the workpiece in the clamping device.

The “8:1 rule”, described also in [28], is also used during milling. This principle recommends milling wall of 1 mm thickness with depth of cut *a_p_* of 8 mm or less. The use of stiffening ribs on the side opposite to the surface to be machined is also advised. They have to be removed at the end of the cutting process. In industrial practice, it is important to gain experience in the machining of thin walls by trial and error, which unfortunately generates significant costs [29]. Another paper [30] presents the simulation of results showing that the application of variable values of technological parameters in individual passes of the tool increases the dimensional and shape accuracy and improves the quality of the machined surface. The authors of papers [17] described that it is possible to minimize the deformations of thin-walled elements made of aluminum alloys after milling thanks to the use of an appropriate machining strategy, pretreatment, and taking into account the technological history of the semifinished product. The doctoral dissertation [31] describes the milling of thin walls at their full height, which is possible by using tools with appropriate stiffness. The full-height machining of thin walls is increasingly recommended and this method was adopted for finishing in the present paper.

Considering the state-of-the-art in manufacturing technology, milling appears to be the most promising machining process with the widest range of applications and at the same time with very high productivity, which is important for economic reasons. The development potential of milling is also evidenced by emerging new milling technologies, improving the quality and efficiency of machining. Their application enables milling to be used in new fields, such as the machining of thin-walled components. Recently, there were two ways of developments in milling: high-speed cutting (HSC) and high-performance cutting (HPC) [32,33,34,35,36]. In the machining of thin-walled workpieces, the application of the HSC method seems to be particularly advantageous, as it is characterized by a reduction in the cutting force after exceeding a certain limit value of the cutting speed *v_c_*. Lower cutting force translates into lower workpiece deformation generated during machining. The value of the cutting force also depends on the properties of the workpiece material, as described in this study, using selected aluminum alloys as an example. In the case of using HSC and HPC technologies for the production of pocket thin-walled elements from monolithic rolled plates, the chips sometimes constitute even more than 95% of the semifinished product weight [37,38,39]. The optimalization of machining process in aspect of its efficiency depends on the use of machining fluids. Currently, efforts are being made to minimize wet cutting, mainly for environmental reasons, which is why minimum quantity cooling lubrication (MQCL) and minimum quantity lubrication (MQL) are increasingly being used. These methods have numerous advantages in comparison to dry cutting, such as improvement of the surface quality, reduction of the tool wear, and machining temperature [40,41].

The aim of the study was a comparison of the geometrical accuracy of thin-walled elements made of different aluminum alloys depending on their mechanical properties as well as cutting speed used during machining.

## 2. Materials and Methods

**Figure 2:**
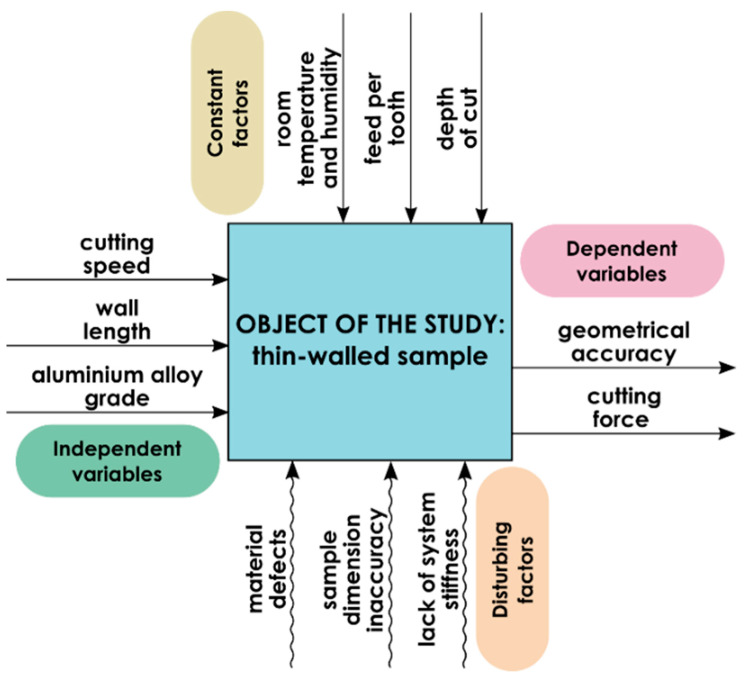
Test object model containing independent and dependent variables as well as constant and disturbing factors [17].

A model of the test object, along with the factors affecting it and the output data analyzed, is shown in Figure 2. The object of the study was thin-walled sample and the research dealt with the geometry of thin walls with different lengths. The independent variables were cutting speed, wall length, and aluminum alloy grade, while the dependent variables include geometrical accuracy and cutting force. The constant factors were technological parameters such as: feed per tooth and depth of cut, as well as room temperature and humidity. The disturbing factors involve lack of system stiffness, material defects, and simple dimension inaccuracy.

Firstly, strength tests were carried out to experimentally confirm the properties of the materials.

The components tested were made from following aluminum alloy grades with significantly different properties:

- EN AW-6082 T651—wrought aluminum alloy;
- EN AW-7075 T651—wrought aluminum alloy;
- EN AC-43000—cast alloy.

The applied wrought materials were heat treated by precipitation hardening. The semifinished products made of wrought alloys were used in the form of rolled plates with thickness of 2 inch (50.8 mm) and cast alloy as cast block with thickness of 70 mm (pre-milled for 50.8 mm).

In terms of machinability, aluminum alloys are divided into 3 basic groups [42]. Therefore, one alloy was selected for the study as a representative of a given group:

- Group-I—pure aluminum and low-alloy wrought aluminum alloys—EN AW-6082 T651 alloy;
- Group-II—strain-hardened or precipitation-hardened wrought aluminum alloys—EN AW-7075 T651 alloy;
- Group-III—cast aluminum alloys with high content of silicon (Si)—EN AC-43000 alloy.

The selected alloys have different mechanical properties, and they are used in industry to produce various structures. The choice of such materials was aimed at facilitating the verification of the influence of their properties on the accuracy of the manufactured elements.

Table 1 presents chemical composition of tested aluminum alloys, while the theoretical mechanical properties are listed in Table 2.

**Figure 3:**
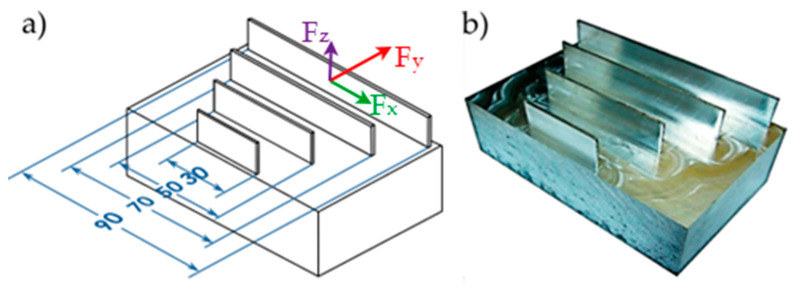
Views of: (**a**) sample model, (**b**) machined sample (all dimensions in mm).

**Figure 4:**
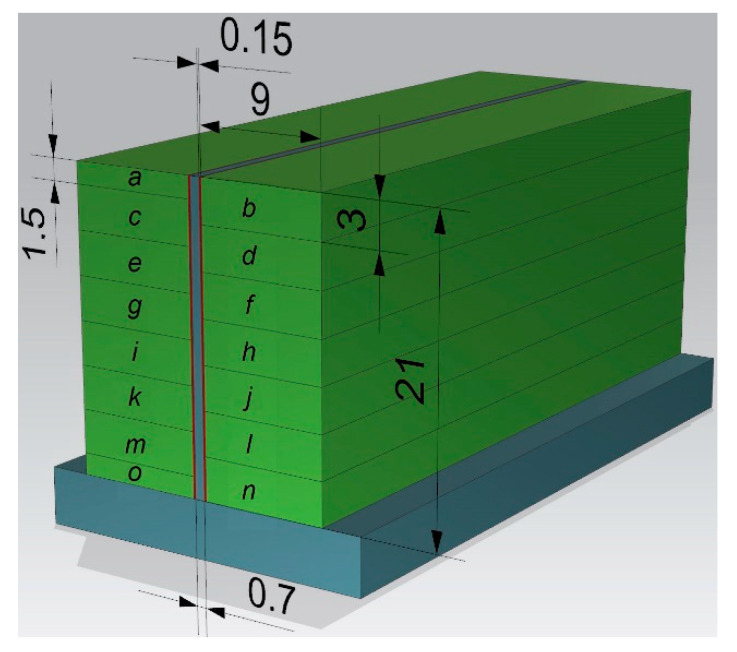
Schematic of wall machining strategy: a-o—tool transitions (all dimensions in mm).

Geometrical views of the samples are shown in Figure 3. The machined walls had a thickness of *t* = 0.7 mm, while their lengths *l* totalled 30, 50, 70, and 90 mm, respectively. All walls had a height of *h* = 21 mm, giving a ratio of *h*/*t* = 30. The selected sample geometry is a simplification of the actual structures used in industry. The “unrestrained” walls are frequently encountered structures used in portions of pocket elements. Moreover, the current trend related to the tendency to reduce the mass of manufactured parts forces the value of the wall thickness to be lowered, hence we analyzed the wall thickness of 0.7 mm. During roughing milling, the strategy of alternating machining of the wall sides with an offset depth of each transition was used. An allowance of 0.15 mm was left for finishing, which was removed in a single pass, i.e., finishing milling was conducted at full wall height (*a_p_* = *h* = 21 mm). The applied machining strategy is shown schematically in Figure 4 and the tool transitions are marked as a-o.

**Figure 5:**
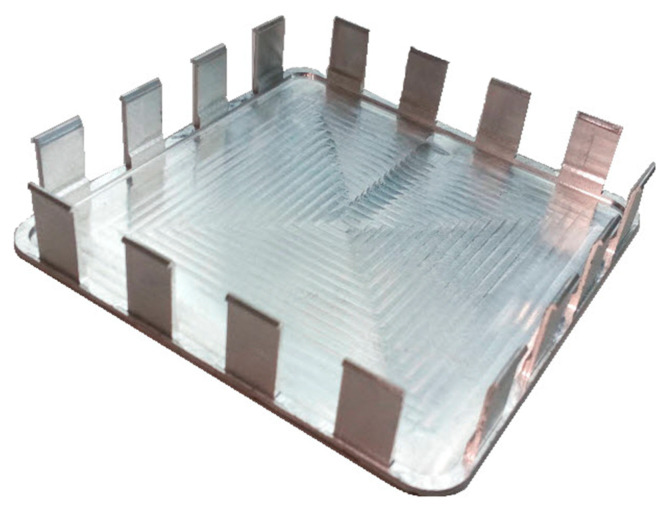
A simplified element of a snap connection in aircraft skin using the geometry of tested walls.

Figure 5 presents an exemplary and simplified element of a snap connection in aircraft skin using the geometry of tested walls.

**Figure 6:**
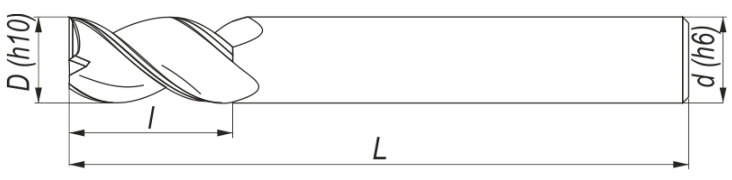
Drawing of milling cutters used. *D*—working part diameter, *d*—clamping part diameter, *l*—maximum depth of cut, *L*—overall length.

The thin-walled workpieces were machined on Avia VMC 800HS vertical-milling center (Warsaw, Poland) by in-cut milling. During the tests, cutting speeds *v_c_* in the HSC range were used, and therefore it was necessary to apply tools that are dedicated for this type of machining and can be used for aluminum alloys belonging to the ISO N material group. For roughing a 2P232-1200-NA H10F cutter of Sandvik (Stockholm, Sweden) with a diameter of 12 mm and two blades was used, while for finishing a 12×22×100-45° W-Z2 of Fenes (Siedlce, Poland) also with a diameter of 12 mm and two blades was used. Figure 6 presents the milling cutter, while Table 3 shows their technical parameters.

**Figure 7:**
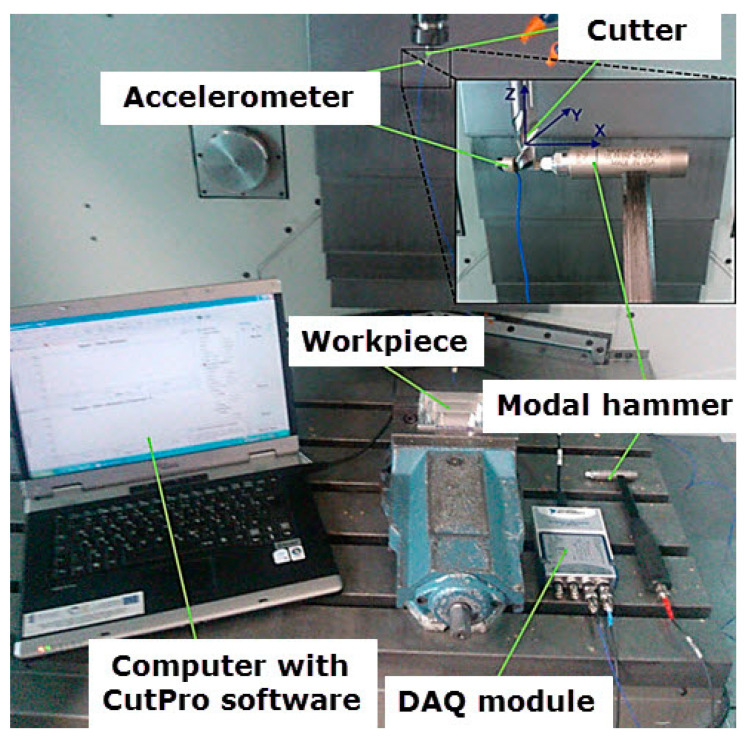
Set for modal analysis consisting of modal hammer, accelerometer, DAQ module, and computer with CutPro software.

Tool holders with spring sleeve in ER standard and a double tool taper HSK63A of Haimer (Hollenbach, Germany) were used for mounting the cutters. It ensures a stable mounting in the machine tool spindle. To guarantee maximum stability, the holders were balanced in the G2.5 class at 25,000 rpm in accordance with [48]. Avia VMC 800HS vertical-milling center (Warsaw, Poland) is equipped with an electro-spindle with a maximum rotational speed of *n* = 24,000 rpm, which enabled the high-speed cutting. Before starting the appropriate cutting tests, the dynamic characteristics of the tool-holder-spindle system was determined for the Fenes cutter used for finishing milling. This research allowed for the established of the stiffness of the above system and the stability areas of its operation. The dynamic characteristics of the tool-holder-spindle system were determined in two directions X and Y, by the method of experimental modal analysis with the use of the set presented in Figure 7. This method measures the value of the exciting force and the response of the system to this excitation, which allows for the determination of the frequency characteristics. The most important elements of the set are a modal hammer with a force measurement sensor and an accelerometer. Signals from these devices go to the data acquisition module, from where, after being converted into a digital signal, they are sent to a computer with CutPro software (9, Vancouver, BC, Canada) for data collection and analysis. During data processing in the CutPro program, the properties of the machined material, technological parameters and tools are defined. On the basis of the collected signals and the defined parameters, the program creates stability curves, which are used to determine the stability areas. In practice, on their basis, it is possible to determine the theoretical values of the depth of cut *a_p_* and rotational speed *n*, ensuring stability of the tool-holder-spindle system.

The roughing and finishing technological parameters are summarised in Table 4. The finishing machining, aimed at comparing the accuracy of thin-walled parts made using different methods, was conducted in two variants, i.e., at a cutting speed of *v_c_* = 300 m/min, corresponding to the conventional machining of aluminum alloys, and at a speed of *v_c_* = 900 m/min, suitable for HSC machining.

The values of the basic strength parameters of the materials used in the study were determined using a static tensile test in accordance with PN-EN ISO 6892-1:2016-09 [49]. The tensile tests were performed using a ZWICK/ROELL Z150 testing machine (Ulm, Germany) with extensometer according to PN-EN ISO 9513:2013-06 [50]. The tests were repeated 10 times for each material.

**Figure 8:**
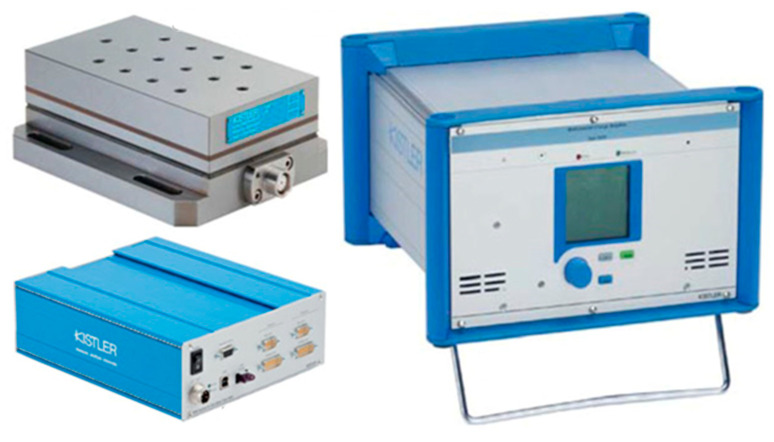
Set for cutting force components measurement.

To determine the range of cutting speeds corresponding to HSC machining, the components of cutting force *F_x_*, *F_y_*, *F_z_* were measured using a Kistler 9257B piezoelectric dynamometer (Winterthur, Switzerland) connected to 5070A charge amplifier. Next, the signal was sent to 5697A DAQ module and finally, it was analyzed in the DynoWare software (Winterthur, Switzerland) (Figure 8). The cutting force components *F_x_*, *F_y_*, *F_z_* (indications according to Figure 3b) were measured for the following cutting speed *v_c_* = 150, 300, 450, 600, 750, and 900 m/min.

**Figure 9:**
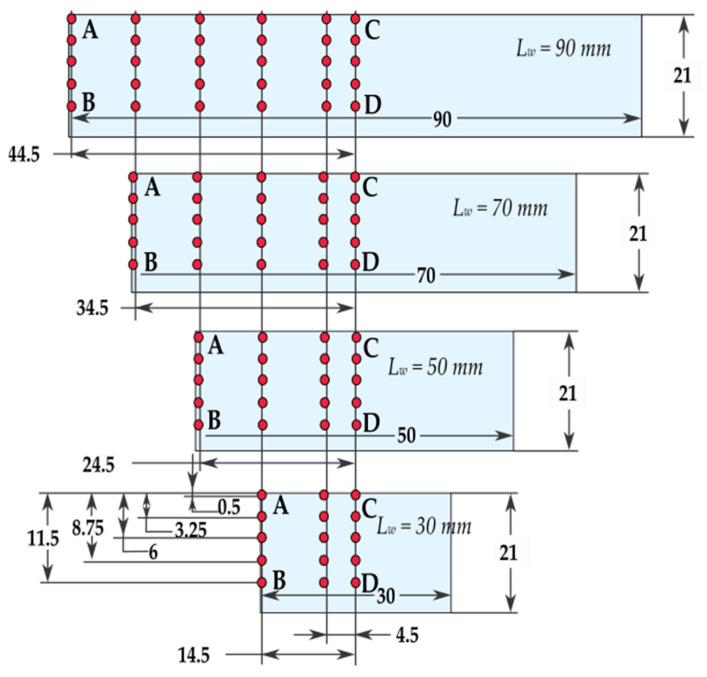
Scheme of test points used during measurements of geometric accuracy (all dimensions in mm).

Measurements of the geometric accuracy of the machined walls were carried out on a Zeiss Vista coordinate measuring machine (Oberkochen, Germany) equipped with a Renishaw PH10 probe head (Wotton-under-Edge, United Kingdom) and a low-pressure TP20 module. Additionally, Figure 9 indicates the location of the measuring points on each wall. Measurements were conducted in several cross-sections, but the following cross-sections were key ones, i.e., extreme cross-section AB and middle cross-sections CD.

The test results were analyzed statistically. The level of significance *α* = 0.05 was adopted and the procedure consisting in comparing two samples with quantitative independent variables was applied. In the first step, it was checked whether the distribution of the examined variables is subject to the normal distribution. For this purpose, the Shapiro-Wilk test was used (it was confirmed that all variables were in accordance with the normal distribution). Next, the analysis of the equality of variances was performed using the Fisher–Snedecor *F*-test. When the null hypothesis *H*_0_ of the variance equality was confirmed, the Student’s *t*-test was used to confirm the equality of means. If the null hypothesis *H*_0_ about the equality of variances was rejected, the Cochran–Cox *C* test was used instead of the Student’s *t*-test (it was not presented in this paper).

After confirming compliance with the normal distribution, the hypothesis of equal variances was verified using the Fisher–Snedecor *F*-test (Equation (1)), assuming the following hypotheses:

- Null hypothesis *H*_0_: $\sigma_{1}^{2}=\sigma_{2}^{2}$;
- Alternative hypothesis *H*_1_: $\sigma_{2}^{2}<\sigma_{1}^{2}$.

(1) $$F=\frac{S_{1}^{2}}{S_{2}^{2}},$$

where: $S_{1}^{2}$—the variance of greater value, $S_{2}^{2}$—the variance of the smaller value.

The calculated value of the *F* was compared with the critical value $F_{cr\left(\alpha ,\;f_{1},\;f_{2}\right)}\;$determined on the basis of the adopted significance level *α* and the number of degrees of freedom *f*_1_ and *f*_2_ determined for analyzed trials (Equations (2) and (3)):

(2) $$f_{1}=n_{1}-1,$$

(3) $$f_{2}=n_{2}-1.$$

The critical area was defined as one-sidedly closed (Equation (4)):

(4) $$O_{cr}=\langle F_{\left(\alpha ,f_{1},\;f_{2}\right)},+\infty ).$$

If the value of the *F* was lower than the critical value *F_cr_* (*F* < *F_cr_*), it meant that it was outside of the critical area and there was no reason to reject the null hypothesis *H*_0_.

Then, when the null hypothesis of the equality of variances *H*_0_ was confirmed, the Student’s *t*-test was used to test the hypothesis of equality of mean values (Equation (5)) in accordance with the hypotheses:

- Null hypothesis *H*_0_: *u_1_* = *u*_2_;
- Alternative hypothesis *H*_1_: *u*_1_ ≠ *u*_2_.

(5) $$t=\frac{\bar{X_{1}}-\bar{X_{2}}}{\sqrt{\frac{n_{1}S_{1}^{2}+n_{2}S_{2}^{2}}{n_{1}+n_{2}-2}\left(\frac{1}{n_{1}}+\frac{1}{n_{2}}\right)}},$$

where: $\bar{X_{1}}$, $\bar{X_{2}}$—mean values, *S_1_*, *S*_2_—variances, *n*_1_, *n*_2_—sample size.

The value of the *t* was compared with the *t_cr_* that was read from table and was determined on the basis of the assumed significance level *α,* and the calculated number of degrees of freedom *f* (Equation (6)):

(6) $$f=n_{1}+n_{2}-2.$$

The critical area was defined as two-sided (Equation (7)):

(7) $$O_{cr}=(-\infty ,-t(\alpha ,f)\rangle \cup \langle t\left(\alpha ,f\right),+\infty ).$$

If the value of the *t* belonged to the critical area, the null hypothesis *H*_0_ was rejected in favor of the alternative hypothesis *H*_1_.

## 3. Results

### 3.1. Mechanical Properties

**Figure 10:**
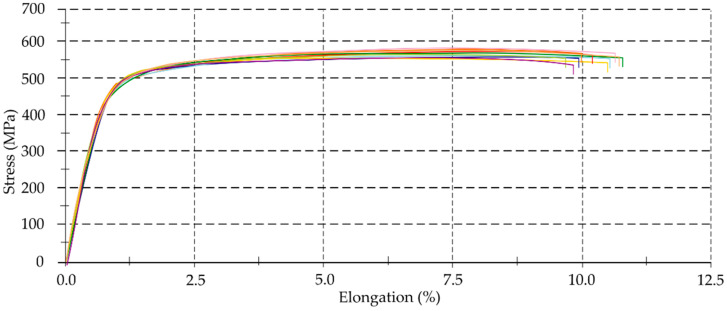
Material characteristics of EN AW-7075 T651 alloy.

**Figure 11:**
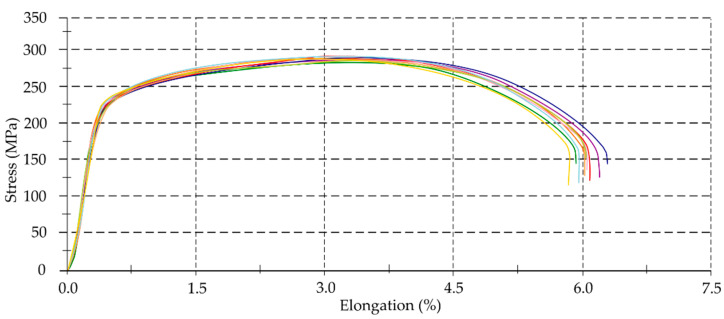
Material characteristics of EN AW-6082 T651 alloy.

**Figure 12:**
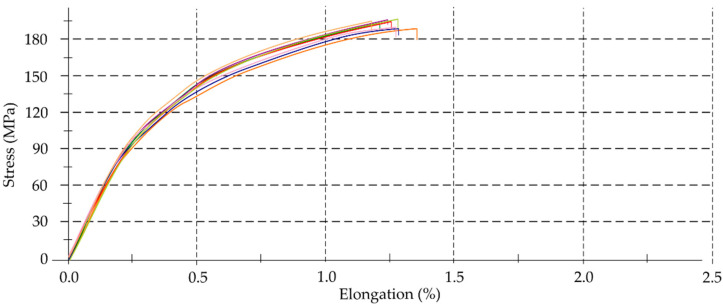
Material characteristics of EN AC-43000 alloy.

The study assumed that the accuracy of the thin-walled components would depend on their geometric, technological parameters and the properties of the workpiece material. The information often provides by manufacturers and contains in standards is not consistent with the values of specific parameters in reality. To identify real values of selected mechanical properties of the alloys used, tensile tests were performed. Figure 10, Figure 11 and Figure 12 present material characteristics of EN AW-7075 T651, EN AW-6082 T651 and EN AC-43000 alloys.

**Figure 13:**
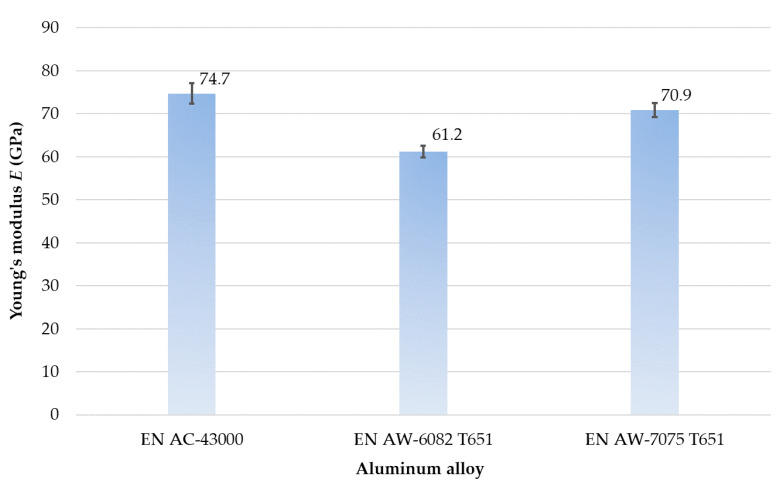
Young’s modulus of aluminum alloys tested.

**Figure 14:**
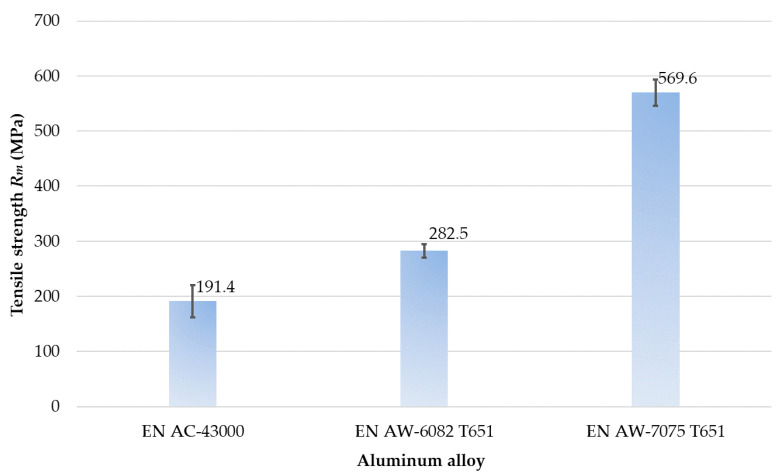
Tensile strength of aluminum alloys tested.

The results of strength research are presented in Table 5 as well as Figure 13 and Figure 14.

Analyzing Young’s modulus *E* (Figure 13), it was found that its greatest value was obtained for the EN AC-43000 cast alloy and the lowest one for the EN AW-6082 T651 wrought alloy. In the case of the EN AW-7075 T651, Young’s modulus *E* was lower by approximately 5% and for the EN AW-6082 T651 by about 18% (in relation to the cast alloy).

Regarding the tensile strength *R_m_* (Figure 14), the greatest value of this parameter was obtained for the EN AW-7075 T651, while the lowest for the EN AC-43000. Comparing the EN AW-7075 T651 alloy to other materials, its tensile strength *R_m_* was over 100% greater than for the EN AW-6082 T651 and approximately 200% compared to that of the EN AC-43000.

Statistical testing was performed only for the Young’s modulus *E*, as unambiguous differences were found for the tensile strength *R_m_*. The results of the statistical verification for the Young’s modulus *E* are presented in Table 6.

On the basis of the results (Table 6), it was found that the values of Young’s modulus for these alloys are different at the adopted significance level α = 0.05.

### 3.2. Modal Analysis

**Figure 15:**
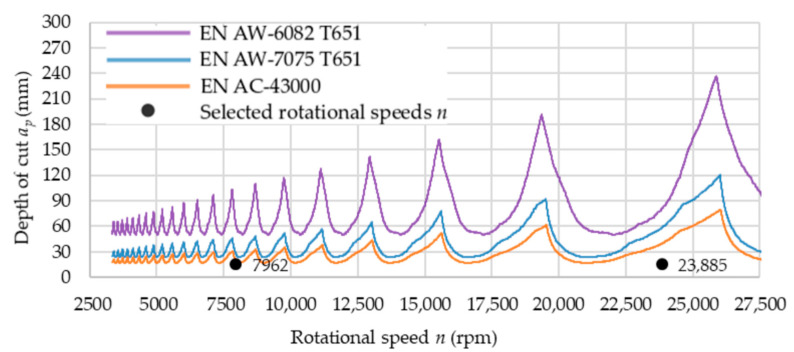
Stability chart for Fenes milling cutter used for finishing machining.

As part of the research, a modal analysis was carried out to determine stable areas of milling with the use of milling cutter of Fenes (dedicated for finishing machining). The technological parameters such as: milling width *a_e_* and feed per tooth *f_z_* were defined in software. In addition, the geometry of the tool was given and the type of material was selected. The stability lobes showed the dependence between the rotational speed *n* and the depth of cut *a_p_*, defining the stable and unstable areas. It is very important information, especially during machining of thin-walled components. Figure 15 presents stability chart for Fenes milling cutter used for finishing machining. Additionally, the selected rotational speeds *n* were marked (*n* = 7962 rpm—conventional machining, *n* = 23,885 rpm—HSC).

Analyzing the stability lobes, it is possible to observe the existence of a limit value of the depth of cut *a_p_* below which, theoretically, a stable machining will always be ensured, regardless of the rotational speed *n* used. On the basis of the chart (Figure 15), the depth of cut *a_p_* is the highest for the EN AW-6082 T651 alloy. The stability lobes for this alloy are displaced upwards in comparison to that of the other materials. It increases the tool stability areas. For the EN AW-7075 T651 and EN AC-43000 alloys, the designated stability areas assume similar values.

### 3.3. Cutting Force

**Figure 16:**
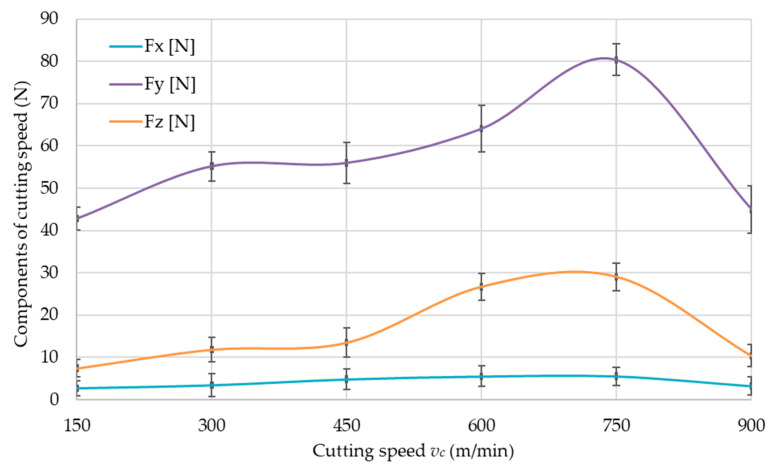
Components of cutting force as a function of cutting speed *v_c_* for EN AW-6082 T651 aluminum alloy.

**Figure 17:**
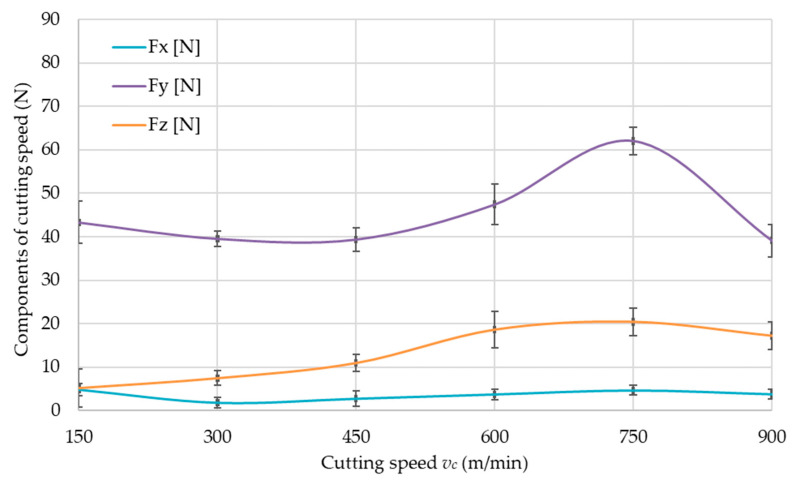
Components of cutting force as a function of cutting speed *v_c_* for EN AC-43000 aluminum alloy.

**Figure 18:**
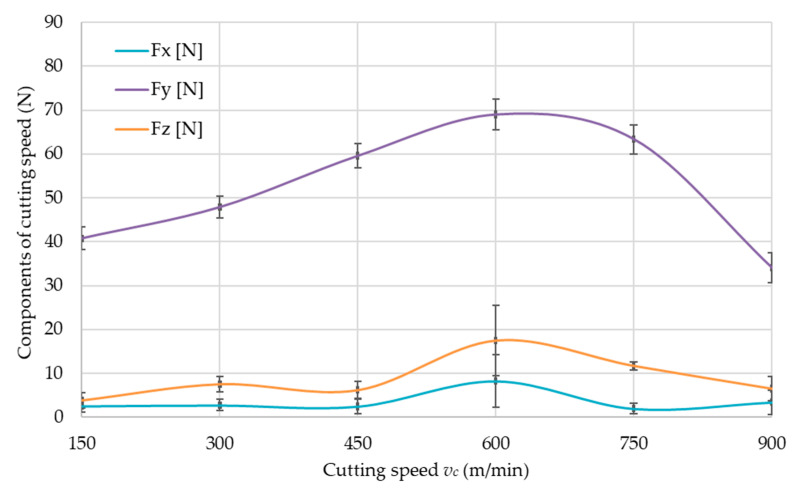
Components of cutting force as a function of cutting speed *v_c_* for EN AW-7075 T651 aluminum alloy.

The values of cutting force components, i.e., *F_x_*, *F_y_* and *F_z_*, for the tested aluminum alloys are shown in Figure 16, Figure 17 and Figure 18. The cutting force components are presented as a function of the cutting speed *v_c_*, to determine the limiting cutting speed *v_c_*, which is the boundary between conventional machining and HSC. Therefore, the charts show characteristic maximum values for all materials. The boundary cutting speed *v_c_* strongly depends on the properties of the machined material and it is clearly visible in the Figure 16, Figure 17 and Figure 18.

On the basis of the obtained results (Figure 16, Figure 17 and Figure 18), it was unequivocally stated that the greatest values were obtained for the *F_y_* component in comparison to other components. The lowest values were recorded for *F_x_* component. The presented relationships were confirmed for all tested materials.

**Figure 19:**
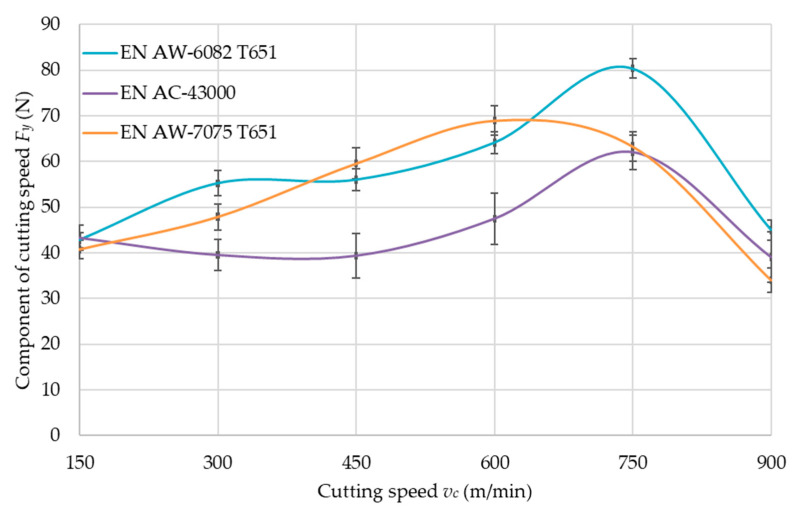
Component of cutting force *F_y_* as a function of cutting speed *v_c_* for tested aluminum alloys.

An accurate analysis was carried out for the component of the cutting force *F_y_*, for which the greatest values were obtained compared to the other components. This component acts perpendicular to the machined wall. The values of *F_y_* as a function of cutting speed *v_c_* for tested aluminum alloys are presented in Figure 19.

Based on the results obtained (Figure 19), a noticeable decrease in the component of cutting force *F_y_* was found for the EN AW-7075 T651 during *v_c_* = 750 m/min and for the EN AW-6082 T651 and EN AC-43000 at *v_c_* = 900 m/min. This means that it is a boundary between conventional and high-speed machining. To ensure that the machining of the walls takes place in the HSC range, a cutting speed of *v_c_* = 900 m/min was employed for this machining method.

Statistical testing was performed for the component of cutting force *F_y_*. The results of the statistical verification are presented in Table 7 and Table 8.

On the basis of the results (Table 7 and Table 8), it was found that the variances were equal in all cases. Verifying the hypothesis of the equality of mean values, during the comparison of EN AC-43000 and EN-AW 7075 T651, the hypothesis about the equality of means was adopted. For the remainder, the inequality of these values was noted. Thus, it was found that the type of material used affects the value of the cutting force component *F_y_*. Moreover, the inequality of the means was found in the case of comparing the cutting speeds *v_c_* = 600 m/min and *v_c_* = 750 m/min for EN AW-7075 T651 alloy.

### 3.4. Geometrical Accuracy

**Figure 20:**
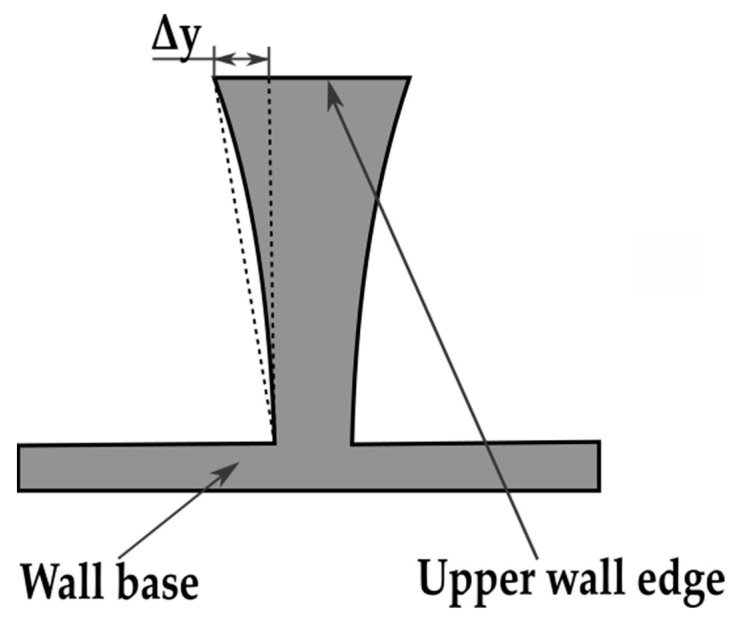
Scheme of thin-walled wall deformation. ∆*y*—wall deformation.

Figure 20 shows schematically the deformation of a thin-walled wall. In all cases, the walls had a trapezoidal shape. They were wider in the upper part (with greater thickness) and narrower in the lower part (with less thickness). The deformation Δ*y* assumed in the charts is the difference between the thicknesses at the top wall and at the bottom of wall.

**Figure 21:**
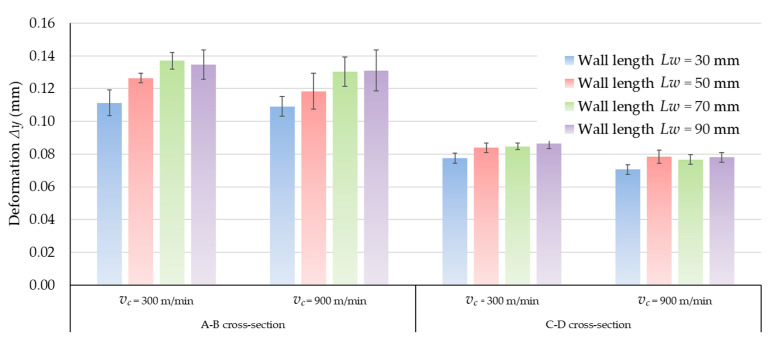
Wall deformations of the AW-7075 T651 alloy.

Figure 21 presents the values of the wall deformations in the extreme and central sections, depending on their length for AW-7075 T651 alloy. For all the materials used, it can be observed that the deformations on the middle cross-sections of the walls (cross-section C-D according to Figure 9) are of a similar value, regardless of their length. Only for the wall with the shortest length of 30 mm they are slightly lower. The deformation values increase with increasing cross-section distances from the wall center. However, the changes are not proportionate to the distance of the cross-section from the center of the wall. In the middle of the walls, a zone can be observed for which the deformation does not change. The greatest deformations of the walls occur in the outermost cross-sections (cross-section A-B according to Figure 9). The deformations in these cross-sections increases with increasing wall length in the range 30 mm to 70 mm. For wall lengths of 70 and 90 mm, it can be assumed that the values of strain in the outermost cross-sections are practically equal. Similar relations were obtained for the other two materials.

**Figure 22:**
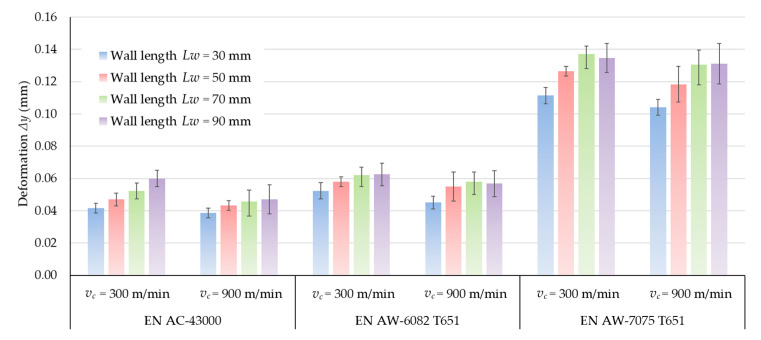
Deformation of walls made of different aluminum alloys of A-B cross-section.

Figure 22 shows the results of measurements of wall deformations in extreme cross-sections A-B made of three selected aluminum alloys. Comparing these results, the walls made of the EN AW-7075 T651 alloy undergo the greatest deformations during machining. They are almost three-times greater than deformations on walls made of the other two materials. The walls made of the EN AC-43000 cast alloy undergo the least elastic deformation. The deformations of the walls made of the EN AW-6082 T651 alloy present similar values to those obtained for the EN AC-43000 alloy. The values of walls deformations at the cutting speed *v_c_* = 900 m/min are lower than for the speed *v_c_* = 300 m/min for all range of wall length. The difference is approximately 10% (in relation to *v_c_* = 900 m/min) for tested materials.

**Figure 23:**
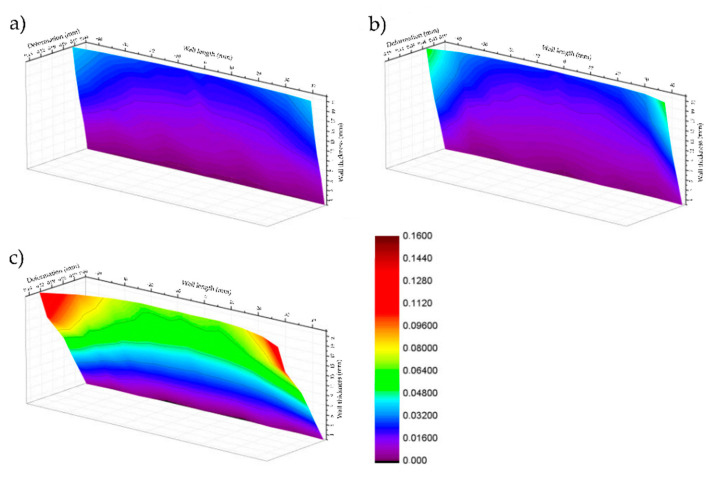
Deformation of thin walls distribution on their length (*L_w_* = 90 mm) and height obtained at cutting speed of *v_c_* = 900 m/min for: (**a**) EN AC-43000, (**b**) EN AW-6082 T651 and (**c**) EN AW-7075 T651.

Figure 23 presents, in the form of 3D charts, the deformation of thin walls distribution on their length (*L_w_* = 90 mm) and height obtained at cutting speed of *v_c_* = 900 m/min, corresponding to the tested materials: EN AC-43000, EN AW-6082 T651 and EN AW-7075 T651, respectively. On the basis of the presented results, it was confirmed that for a wall with a length of *L_w_* = 90 mm and machined at *v_c_* = 900 m/min, the greatest deformation was noted for sample made of the EN AW-7075 T651 wrought alloy as well as the smallest one for the EN AW-43000 cast alloy. Moreover, it is noticeable that the greatest deformations were obtained at the ends of the walls.

The statistical analysis was made for this research. The results of selected tests are presented in Table 9, Table 10, Table 11, Table 12, Table 13 and Table 14.

Based on the statistical analysis (Table 9, Table 10, Table 11, Table 12, Table 13 and Table 14), it was found that in all cases the equality of variances was obtained, but the hypothesis of equality of means was rejected, hence the conclusion that the deformations generated depend on the type of material, wall length, and the cutting speed *v_c_* used. The results for the cross-sections A-B and C-D were not compared, as it was unequivocally found that the obtained values of deformation were different.

## 4. Discussion

### 4.1. Mechanical Properties

On the basis of results of mechanical properties (Table 5, Figure 10, Figure 11, Figure 12, Figure 13 and Figure 14), it can be concluded that the mechanical properties of the aluminum alloys studied varied considerably. Particularly significant differences can be observed between the EN AC-43000 cast alloy and the EN AW-6082 T651 as well as the EN AW-7075 T651 wrought alloys. This alloy (EN AC-43000), with the highest Young’s modulus *E*, has the lowest tensile strength *R_m_*. From the results obtained, it is a “fragile” alloy, with high stiffness, which is characteristic of cast Al-Si alloys. The tested wrought alloys also exhibit significant differences in their mechanical properties. The EN AW-6082 T651 alloy has lower almost twice the tensile strength and more than 1.5-times relative elongation, compared to the EN AW-7075 T651 alloy. Furthermore, this alloy has the lowest Young’s modulus of the three materials compared. The obtained values of the selected mechanical properties of this alloy confirmed its high ductility. To highlight the differences in the accuracy of thin-walled components, alloys with significantly different mechanical properties were deliberately used in the study.

### 4.2. Cutting Force

In accordance with the data regarding component of cutting force *F_y_*, the cutting speed *v_c_* corresponding to the maximum value of the cutting force *F_y_* can be taken as the boundary between conventional and HSC machining, as stated in the introduction. The value of the cutting-speed limit strongly depends on the properties of the workpiece material, as can be seen in Figure 16, Figure 17 and Figure 18. The EN AW-6082 T651 and the EN AC-43000 alloys with “poorer” mechanical properties in comparison to the EN AW-7075 T651 alloy have a higher value of the cutting-speed limit. This means that for these two alloys, a cutting speed *v_c_* = 900 m/min has to be used to achieve machining conditions corresponding to HSC. For the EN AW-7075 T651 alloy, the limiting cutting speed is lower, at approx. *v_c_* = 750 m/min. Based on the authors’ own observations and literature data, it can be concluded that the value of the limiting cutting speed *v_c_* for a given alloy does not depend on the parameters describing the cross-section of the machined layer, i.e., milling width *a_e_*, depth of cut *a_p_*, and feed per tooth *f_z_*.

### 4.3. Geometrical Accuracy

The EN AW-7075 T651 alloy is a material of high elasticity and strength. Due to its high elasticity, walls made of this material undergo significantly more elastic deformation during milling than those made of the other materials. After the transition of the tool, their “elastic return” occurs, and significant wall thickening is obtained at its upper edge. The walls made of the EN AC-43000 cast alloy undergo the least elastic deformation. This alloy has a similar Young’s modulus as the EN AW-7075 T651 alloy but is characterized by much greater “fragility” as well as lower cutting resistance, which is characteristic of cast alloys and translates into lower cutting forces. These factors ensure that during machining the elastic deformation on the walls made of this alloy is low, which translates into a reduction in the shape errors obtained. The deformations of the walls made of EN AW-6082 T651 alloy present similar values to those obtained for the EN AC-43000 alloy. The EN AW-6082 T651 alloy, due to its low content of alloying elements, exhibits the highest plasticity among the three materials tested. The low elasticity of this material (the lowest Young’s modulus of the alloys studied) means that it undergoes only minor elastic deformation during machining, which translates into a slight deformation of the walls made thereof. However, when machining this alloy, the highest cutting forces occur within almost the whole range of the analyzed cutting speeds *v_c_*, caused by the difficult decohesion of group I aluminum alloys. The machining process of alloys of this group can temporarily transform into burnishing, which, in combination with high ductility, adhesion to the tool material and susceptibility to strain hardening of alloys of this group, results in increased cutting forces. The EN AW-7075 T651 wrought alloy is characterized by the highest yield point compared to that of the other materials. Therefore, the yield point is probably a property of the material also influencing its deformability in the case of the production of thin-walled elements.

The values of wall deformation at the cutting speed *v_c_* = 900 m/min are lower than for the speed *v_c_* = 300 m/min. This confirms the suitability of the HSC method for machining thin-walled components, especially as its application also increases machining productivity.

## 5. Conclusions

The study led to the following:

- The deformation values increase with increasing cross-section distances from the wall center and the greatest deformations of the walls occur in the outermost cross-sections. The ends of the walls are the most subject to deformation. Moreover, the deformation grows also with increasing wall length. The greatest deformation occurs at length of 90 mm, while the smallest one at length of 30 mm.
- The samples made of EN AW-7075 T651 alloy undergo the greatest deformations during machining in comparison to that of other two materials (EN AW-6082 T651 and EN AC-43000). The differences in deformations of thin-walled elements made of different aluminum alloys mainly result from significantly various mechanical properties of the tested materials.
- The lowest values of wall deformation occur at the cutting speed *v_c_* = 900 m/min (corresponds to high-speed cutting) as well as the biggest one at the cutting speed *v_c_* = 300 m/min (corresponds to conventional machining) for all analyzed configurations.
- An increase in the geometrical accuracy of the manufactured elements is also observed with the increase in the cutting speed for the HSC range. It is also related to the reduction of the cutting force after exceeding the limiting cutting speed, which is the boundary between conventional machining and HSC. The values of the components of the cutting force and limiting cutting speed differed depending on the material tested. In addition, the selection of technological parameters was preceded by a modal analysis, on the basis of which the stable areas of the tool’s operation were selected.
- To minimize the postmachining deformation of thin-walled components, the use of high-speed HSC machining is justified, particularly with a view to also increasing machining productivity in comparison to that of conventional machining and related material removal rate per unit time, i.e., rate of metal removal.

## Figures and Tables

**Table 1:** Chemical composition of aluminum alloys [43,44].

Wrought Aluminum Alloys	Chemical Composition (%)										
	Si	Fe	Mg	Cu	Mn	Zn	Ti	Cr	Other	Al	
EN AW-6082 T651	1	≤0.5	0.9	≤0.1	0.7	≤0.2	≤0.1	≤0.25	-	Rest	
EN AW-7075 T651	≤0.4	≤0.5	2.5	1.6	≤0.3	5.6	≤0.2	0.23	Zr + Ti ≤0.25	Rest	
**Cast Aluminum** **Alloy**	**Si**	**Cu**	**Mg**	**Mn**	**Cr**	**Fe**	**Ti**	**Zn**	**Ni**	**Pb**	**Al**
EN AC-43000	10	≤0.05	0.33	≤0.45	≤0.1	≤0.55	≤0.15	≤0.1	≤0.05	≤0.05	Rest

**Table 2:** Mechanical properties of aluminum alloys [44,45].

Aluminum Alloys	Mechanical Properties			
	Yield Point *R*_*p*0.2_ (MPa)	Tensile Strength *R_m_* (MPa)	Elongation *A* (%)	Brinell Hardness (HB)
EN AW-6082 T651	240	295	8	89
EN AW-7075 T651	440	525	11	155
EN AC-43000	180	220	1	75

**Table 3:** Technical parameters of cutters used [46,47].

Symbol	Sandvik 2P232-1200-NA H10F	Fenes 12×22×100-45° W-Z2
Number of teeth, *z*	2	2
Working part diameter, *D* (mm)	12	12
Overall length, *L* (mm)	83	100
Maximum depth of cut, *l* (mm)	22	22
Clamping part diameter, *d* (mm)	12	12

**Table 4:** Technological parameters applied during roughing and finishing.

Technological Parameters	Roughing	Conventional Finishing	HSC Finishing
*v_c_* (m/min)	300	300	900
*f_z_* (mm/tooth)	0.15	0.07	0.07
*a_p_* (mm)	3	21	21
*a_e_* (mm)	9	0.15	0.15

**Table 5:** Selected mechanical properties of alloys studied.

Aluminum Alloys	Selected Mechanical Properties			
	Young’s Modulus *E* (GPa)	Yield Point *R*_*p*0.2_ (MPa)	Tensile Strength *R_m_* (MPa)	Elongation *A* (%)
EN AW-6082 T651	61.2	227.4	282.5	6.1
EN AW-7075 T651	70.9	445.8	569.6	10.3
EN AC-43000	74.7	141.1	191.4	1.2

**Table 6:** Statistical verification of results of Young’s modulus (α = 0.05).

Tests	*F*	*F_cr_*	Result	*t*	*t_cr_*	Result
EN AC-43000– EN AW-6082 T651	3.1080	3.1789	$\sigma_{1}^{2}=\sigma_{2}^{2}$	14.8014	2.1010	${µ}_{1}\neq {µ}_{2}$
EN AC-43000– EN AW-7075 T651	2.2127	3.1789	$\sigma_{1}^{2}=\sigma_{2}^{2}$	3.9751	2.1010	${µ}_{1}\neq {µ}_{2}$
EN AW-6082 T651– EN AW-7075 T651	1.4047	3.1789	$\sigma_{1}^{2}=\sigma_{2}^{2}$	13.9006	2.1010	${µ}_{1}\neq {µ}_{2}$

**Table 7:** Statistical verification of results of component of cutting force *F_y_* for analyzed aluminum alloys and *v_c_* = 750 m/min (α = 0.05).

Tests	*F*	*F_cr_*	Result	*t*	*t_cr_*	Result
EN AC-43000–EN AW-6082 T651	1.3491	6.3882	$\sigma_{1}^{2}=\sigma_{2}^{2}$	7.4269	2.3060	${µ}_{1}\neq {µ}_{2}$
EN AC-43000–EN AW-7075 T651	1.0503	6.3882	$\sigma_{1}^{2}=\sigma_{2}^{2}$	0.5789	2.3060	${µ}_{1}={µ}_{2}$
EN AW-6082 T651–EN AW-7075 T651	1.2844	6.3882	$\sigma_{1}^{2}=\sigma_{2}^{2}$	6.8135	2.3060	${µ}_{1}\neq {µ}_{2}$

**Table 8:** Statistical verification of results of component of cutting force *F_y_* for cutting speeds *v_c_* and EN AW-7075 T651 alloy (α = 0.05).

Tests	*F*	*F_cr_*	Result	*t*	*t_cr_*	Result
*v_c_* = 600 m/min–*v_c_* = 750 m/min	1.1638	6.3882	$\sigma_{1}^{2}=\sigma_{2}^{2}$	2.3251	2.3060	${µ}_{1}\neq {µ}_{2}$

**Table 9:** Statistical verification of results of the deformation for tested aluminum alloys at *L_w_* = 30 mm and *v_c_* = 300 m/min (α = 0.05).

Tests	*F*	*F_cr_*	Result	*t*	*t_cr_*	Result
EN AC-43000–EN AW-6082 T651	2.7778	3.1789	$\sigma_{1}^{2}=\sigma_{2}^{2}$	5.4536	2.1010	${µ}_{1}\neq {µ}_{2}$
EN AC-43000–EN AW-7075 T651	2.7778	3.1789	$\sigma_{1}^{2}=\sigma_{2}^{2}$	35.8261	2.1010	${µ}_{1}\neq {µ}_{2}$
EN AW-6082 T651–EN AW-7075 T651	1.0000	3.1789	$\sigma_{1}^{2}=\sigma_{2}^{2}$	25.0457	2.1010	${µ}_{1}\neq {µ}_{2}$

**Table 10:** Statistical verification of results of deformation for tested aluminum alloys at *L_w_* = 30 mm and *v_c_* = 900 m/min (α = 0.05).

Tests	*F*	*F_cr_*	Result	*t*	*t_cr_*	Result
EN AC-43000–EN AW-6082 T651	1.7778	3.1789	$\sigma_{1}^{2}=\sigma_{2}^{2}$	3.8780	2.1010	${µ}_{1}\neq {µ}_{2}$
EN AC-43000–EN AW-7075 T651	2.7778	3.1789	$\sigma_{1}^{2}=\sigma_{2}^{2}$	33.6995	2.1010	${µ}_{1}\neq {µ}_{2}$
EN AW-6082 T651–EN AW-7075 T651	1.5625	3.1789	$\sigma_{1}^{2}=\sigma_{2}^{2}$	27.6599	2.1010	${µ}_{1}\neq {µ}_{2}$

**Table 11:** Statistical verification of results of deformation for tested cutting speeds *v_c_* at *L_w_* = 30 mm and EN AC-43000 (α = 0.05).

Tests	*F*	*F_cr_*	Result	*t*	*t_cr_*	Result
*v_c_* = 300 m/min–*v_c_* = 900 m/min	1.0000	3.1789	$\sigma_{1}^{2}=\sigma_{2}^{2}$	2.2156	2.1010	${µ}_{1}\neq {µ}_{2}$

**Table 12:** Statistical verification of results of deformation for tested cutting speeds *v_c_* at *L_w_* = 30 mm and EN AW-6082 T651 (α = 0.05).

Tests	*F*	*F_cr_*	Result	*t*	*t_cr_*	Result
*v_c_* = 300 m/min–*v_c_* = 900 m/min	1.0000	3.1789	$\sigma_{1}^{2}=\sigma_{2}^{2}$	3.4061	2.1010	${µ}_{1}\neq {µ}_{2}$

**Table 13:** Statistical verification of results of deformation for tested cutting speeds *v_c_* at *L_w_* = 30 mm and EN AW-7075 T651 (α = 0.05).

Tests	*F*	*F_cr_*	Result	*t*	*t_cr_*	Results
*v_c_* = 300 m/min–*v_c_* = 900 m/min	1.0000	3.1789	$\sigma_{1}^{2}=\sigma_{2}^{2}$	3.0830	2.1010	${µ}_{1}\neq {µ}_{2}$

**Table 14:** Statistical verification of results of deformation for tested wall length *L_w_* at *v_c_* = 300 m/min and EN AC-43000 (α = 0.05).

**Tests**	***F***	***F_cr_***	**Result**	***t***	***t_cr_***	**Result**
*L_w_* = 30 mm–*L_w_* = 50 mm	1.7778	3.1789	$\sigma_{1}^{2}=\sigma_{2}^{2}$	3.1800	2.1010	${µ}_{1}\neq {µ}_{2}$
*L_w_* = 30 mm–*L_w_* = 70 mm	2.7778	3.1789	$\sigma_{1}^{2}=\sigma_{2}^{2}$	5.4022	2.1010	${µ}_{1}\neq {µ}_{2}$
*L_w_* = 30 mm–*L_w_* = 90 mm	2.7778	3.1789	$\sigma_{1}^{2}=\sigma_{2}^{2}$	9.3793	2.1010	${µ}_{1}\neq {µ}_{2}$
*L_w_* = 50 mm–*L_w_* = 70 mm	1.5625	3.1789	$\sigma_{1}^{2}=\sigma_{2}^{2}$	2.4363	2.1010	${µ}_{1}\neq {µ}_{2}$
*L_w_* = 50 mm–*L_w_* = 90 mm	1.5625	3.1789	$\sigma_{1}^{2}=\sigma_{2}^{2}$	6.0580	2.1010	${µ}_{1}\neq {µ}_{2}$
*L_w_* = 70 mm–*L_w_* = 90 mm	1.0000	3.1789	$\sigma_{1}^{2}=\sigma_{2}^{2}$	3.2796	2.1010	${µ}_{1}\neq {µ}_{2}$

## Data Availability

The data presented in this study are available on request from the corresponding author.
